# JCPyV-Induced MAPK Signaling Activates Transcription Factors during Infection

**DOI:** 10.3390/ijms20194779

**Published:** 2019-09-26

**Authors:** Jeanne K. DuShane, Colleen L. Mayberry, Michael P. Wilczek, Sarah L. Nichols, Melissa S. Maginnis

**Affiliations:** 1 Department of Molecular and Biomedical Sciences, The University of Maine, Orono, ME 04469, USA; jeanne.dushane@maine.edu (J.K.D.); colleen.mayberry@maine.edu (C.L.M.); michael.wilczek@maine.edu (M.P.W.); sarah.l.nichols@maine.edu (S.L.N.); 2 Graduate School in Biomedical Sciences and Engineering, The University of Maine, Orono, ME 04469, USA

**Keywords:** JC polyomavirus, mitogen-activated protein kinase, extracellular signal-regulated kinase, Raf, cMyc, cJun, SMAD

## Abstract

JC polyomavirus (JCPyV), a ubiquitous human pathogen, is the etiological agent of the fatal neurodegenerative disease progressive multifocal leukoencephalopathy (PML). Like most viruses, JCPyV infection requires the activation of host-cell signaling pathways in order to promote viral replication processes. Previous works have established the necessity of the extracellular signal-regulated kinase (ERK), the terminal core kinase of the mitogen-activated protein kinase (MAPK) cascade (MAPK-ERK) for facilitating transcription of the JCPyV genome. However, the underlying mechanisms by which the MAPK-ERK pathway becomes activated and induces viral transcription are poorly understood. Treatment of cells with siRNAs specific for Raf and MAP kinase kinase (MEK) targets proteins in the MAPK-ERK cascade, significantly reducing JCPyV infection. MEK, the dual-specificity kinase responsible for the phosphorylation of ERK, is phosphorylated at times congruent with early events in the virus infectious cycle. Moreover, a MAPK-specific signaling array revealed that transcription factors downstream of the MAPK cascade, including cMyc and SMAD4, are upregulated within infected cells. Confocal microscopy analysis demonstrated that cMyc and SMAD4 shuttle to the nucleus during infection, and nuclear localization is reduced when ERK is inhibited. These findings suggest that JCPyV induction of the MAPK-ERK pathway is mediated by Raf and MEK and leads to the activation of downstream transcription factors during infection. This study further defines the role of the MAPK cascade during JCPyV infection and the downstream signaling consequences, illuminating kinases as potential therapeutic targets for viral infection.

## 1. Introduction

JC polyomavirus (JCPyV) is a common human pathogen that can cause a fatal neurological disease in individuals who are immunocompromised [1]. JCPyV infects between 50% and 80% of the population and causes a persistent asymptomatic kidney infection in healthy individuals [2,3]. Among individuals who are severely immunosuppressed, often in association with HIV-1 infection or prolonged immunomodulatory therapy treatment for multiple sclerosis (MS), JCPyV reactivates and spreads to the central nervous system (CNS) [4,5,6]. Within the CNS, JCPyV infects glial cells, astrocytes, and oligodendrocytes [7,8] responsible for production of myelin and myelination support. Viral cytolytic destruction of the myelin-producing oligodendrocytes results in the loss of myelin within the CNS resulting in the development of the fatal neurodegenerative disease progressive multifocal leukoencephalopathy (PML) [9,10]. The formation of lesions within the brain is the hallmark of PML onset, and symptoms of the disease include hemiparalysis and altered cognitive functioning [11]. Currently, there are limited therapies available for the treatment or prevention of PML, yielding a high mortality rate for this devastating disease [12].

JCPyV is a small, non-enveloped virus with a double-stranded DNA genome encased within a proteinaceous capsid [12]. Infection of host cells is initiated by JCPyV attachment to the terminal α2,6-linked sialic acid on lactoseries tetrasaccharide c (LSTc) via the exterior viral capsid protein, viral protein 1 (VP1) [12,13,14,15]. This attachment receptor, however, only facilitates viral binding to the host cell; JCPyV requires the 5-hydroxytryptamine 2 (5-HT_2_) family of receptors for entry into the cell [16,17]. Viral internalization into the host-cell is facilitated by clathrin-mediated endocytosis machinery, including the scaffolding protein β-arrestin [18,19]. Upon the completion of endocytosis, the virus traffics through the endocytic compartment to the endoplasmic reticulum (ER), where the virion capsid becomes partially uncoated [20]. JCPyV then translocates into the host-cell nucleus where DNA replication machinery is hijacked by the virus to facilitate transcription and replication of the viral genome in a temporal fashion [21]. The viral early genes, comprised of the T-antigens (TAg), drive the replicative process and the subsequent production of the capsid components, late viral proteins VP1, VP2, and VP3 [12,21]. Temporal regulation of the viral genome is common to nearly all DNA viruses, due in large part to the fact that most DNA viruses rely almost completely on hijacking the requisite machineries from the host cell in order to replicate [22].

An essential part of host-cell dysregulation during viral infection occurs through the manipulation of cellular signaling mechanisms, the cascades that facilitate intracellular and extracellular communication. JCPyV activates the extracellular-signal regulated kinase (ERK) component of the mitogen-activated protein kinase (MAPK-ERK) cascade upon infection, which is required for viral transcription and infection [18,23,24,25]. MAPK-ERK functions to transmit external mitogenic signals inward, to generate the appropriate cellular responses such as: cellular growth, differentiation, or cellular survival [26,27]. The MAPK-ERK pathway proceeds through a series of sequential phosphorylation events of the three main kinases that comprise the MAPK-ERK pathway: Raf, MAP kinase kinase (MEK1/2), and ERK1/2 [26,28]. Initially, the pathway is activated upon ligand binding and receptor tyrosine kinases (RTKs) or G proteins, which then leads to activation of the small GTPase Ras at the plasma membrane [29]. Ras binds to the serine/threonine kinases Raf, of which there are three isoforms, A-Raf, B-Raf, and C-Raf, which phosphorylate MEK1/2 [27,30]. The dual specificity kinases MEK1/2 then phosphorylate ERK1/2 [26,31]. As the terminal kinase in the MAPK-ERK cascade, ERK is a dynamic and potent signaling molecule that activates downstream signaling targets, becomes dephosphorylated by protein phosphatases, activates cytoplasmic targets, or translocates to the nucleus to regulate transcription factors that function in cell proliferation, differentiation, and cell death [31,32].

Activated ERK is linked to over 200 known cellular substrates located in both the cytoplasm and nucleus including MAPK-associated protein kinases, other kinases, and transcription factors that facilitate the transmission of incoming mitogenic signals [26,28,33]. The majority of these downstream effectors enable the transcription and translation of the host-cell gene products necessary for an appropriate response to the initial stimulus. In particular, transcription factors like cMyc, cJun, cFos (AP-1), SMAD4, and others are the predominant transcription factor targets of pERK, eliciting a real-time cellular response to the incoming stimulus [23]. Interestingly, many DNA viruses that rely on hijacking these cellular replication machineries specifically target the MAPK-ERK signaling cascade during the infectious process due the dynamic influence of this cascade over cellular fate. During infection, JCPyV relies on usurping host-cell transcription factors to promote infection including NFAT, NF-kB, SMADs, Sp1, cJun, and cMyc [34,35,36,37,38,39], which are downstream of the MAPK-ERK signaling pathway, suggesting that JCPyV may reprogram host-cell transcriptional machinery through activation of the MAPK-ERK pathway. However, the signaling mechanism(s) that facilitate JCPyV-induced activation of ERK and whether activation of the MAPK-ERK pathway influences the expression of transcription factors necessary for JCPyV infection are currently unclear.

The objective of this study was to determine if JCPyV drives the activation of the canonical MAPK-ERK signaling pathway to promote infection and to determine how host-cell transcription factors respond to ERK activation during infection. At timepoints congruent with viral entry, JCPyV infection induces an upregulation in MEK phosphorylation suggesting that ERK activation occurs through JCPyV-induced activation of MEK. Moreover, key components of the MAPK-ERK cascade, Raf and MEK are required for JCPyV infection as cells treated with Raf- and MEK-specific siRNAs demonstrated a significant reduction in viral infection. These findings suggest that JCPyV infection induces the canonical MAPK-ERK cascade in order to promote infection. Downstream transcription factors targets of both the MAPK pathway and of JCPyV infection including SMAD4 and cMyc localized predominantly in the host-cell nucleus at timepoints associated with viral genome transcription. These data suggest that JCPyV infection may induce the recruitment and activation of MAPK-ERK-associated transcription factors to promote viral gene transcription and successful viral infection. Together, these findings suggest that JCPyV infection activates the MAPK-ERK pathway to drive viral infection through MAPK-ERK activation of downstream transcription factors, which are known to be required for JCPyV infection. 

## 2. Results

### 2.1. Knockdown of Raf Prevents JCPyV Infection

Prior studies have identified that JCPyV activates the MAPK-ERK cascade, specifically ERK, during infection [18,24,25]. However, the mechanism by which ERK becomes activated during JCPyV infection is currently unknown. During canonical host-cell MAPK-ERK signaling, the kinase Raf, directly upstream of both ERK and MEK, serves as the initial kinase of the MAPK-ERK signaling cascade [26]. Activated by Ras-GTP activity, Raf facilitates the propagation of mitogenic signals to MEK and downstream to ERK. As activation of the MAPK-ERK pathway proceeds in a linear fashion, if levels of phosphorylated ERK increase upon JCPyV challenge, it suggests that JCPyV must utilize the kinases upstream of this potent signaling molecule to induce ERK activation, including Raf and MEK. While there are three isoforms of Raf, B- and C-Raf predominantly phosphorylate MEK [40,41]. Further, it has been demonstrated that Bay43-9006, sorafenib, a selective inhibitor of B-Raf and C-Raf (IC_50_ = 22 and 5 nM, respectively) [42], significantly reduces JCPyV infection in SVG-A cells, suggesting B- and C-Raf play an important role in promoting JCPyV infection [25]. However, Bay43-9006 has also been shown to inhibit RTK activity, which is also required for JCPyV infection [18,43]. Due to potential effects of Bay43-9006 inhibition on proteins other than Raf, siRNA knockdown of host-cell Raf proteins was employed as a more specific targeting mechanism for investigating the role of Raf during JCPyV infection.

**Figure 1:**
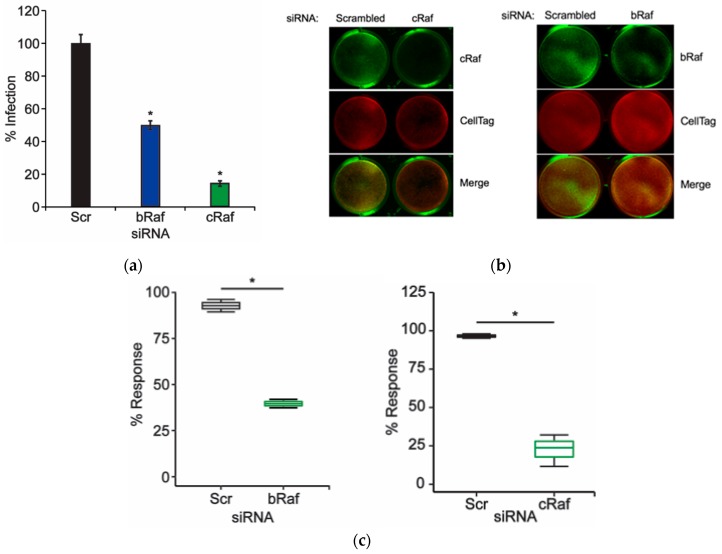
Knockdown of B-Raf and C-Raf inhibits JCPyV infection. SVG-A cells were transfected with either a scrambled (Scr) siRNA control or a B-Raf or C-Raf siRNA and incubated at 37 °C. At 72 h post-transfection, siRNA-transfected cells were either (**a**) infected with JCPyV (MOI: 1 FFU/cell) at 37 °C for 1 h and then fed with cMEM and incubated for 72 h or (**b**,**c**) processed for ICW analysis of protein knockdown using B-Raf or C-Raf antibodies (green) and CellTag control (red). (**a**) Infected cells were fixed and stained to analyze nuclear JCPyV VP1 expression. Data are representative of the mean percentage of JCPyV VP1+ cells per 10x visual field normalized to the siRNA control cells (100%) for triplicate samples (representative of three independent experiments). Error bars = SD. (**b**) B-Raf and C-Raf protein expression was measured for control- and experimental-siRNA treatments by ICW analysis, and (**c**) signal intensity values were quantified per the calculation [(protein/Cell Tag) × 100] as determined through ImageJ analysis. Boxes represent the distribution of the data for three independent experiments; box midline represents the median. Whiskers represent values 1.5 times the distance between the first and third quartiles (inter-quartile range). Student’s *t*-test was used to determine the statistical significance. *, *p* < 0.05.

Based on the selectivity of Bay43-9006 for both B-Raf and C-Raf, the role of both isoforms in JCPyV infection was analyzed using siRNAs specific for B- and C-Raf. SVG-A cells were transfected with either a control, B-Raf-, or C-Raf-specific siRNA, then infected with JCPyV, and infectivity was then scored by indirect immunofluorescence and quantitation of nuclear VP1 expression. Cells treated with B-Raf siRNA demonstrated an ~50% reduction in infectivity in comparison to the control siRNA-treated cells, while C-Raf siRNA-treated cells demonstrated an ~80% reduction in infection (Figure 1A). Protein knockdown by siRNA treatment was analyzed by In-cell Western (ICW) analysis, which demonstrated that B-Raf expression was reduced by ~60% while C-Raf expression was reduced by ~75%. Together, these data suggest that Raf is necessary for promoting successful viral infection, and that both B- and C-Raf play a role in JCPyV infection (Figure 1).

### 2.2. Knockdown of MEK Prevents JCPyV Infection

**Figure 2:**
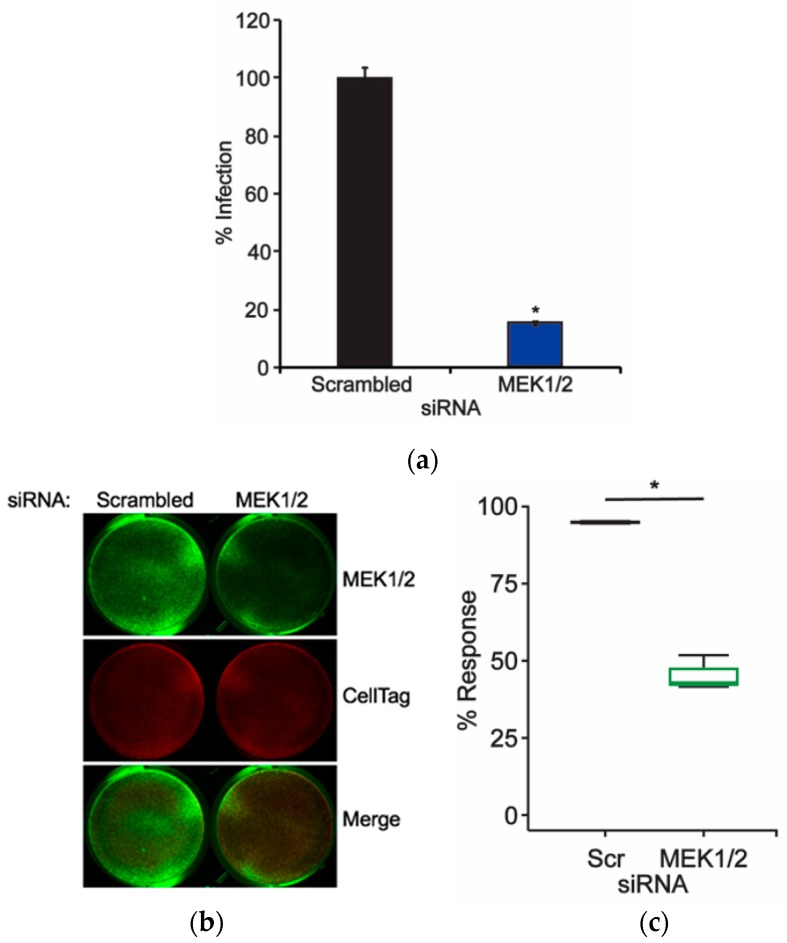
Knockdown of MEK1/2 inhibits JCPyV infection. SVG-A cells were transfected with either a scrambled (Scr) siRNA control or a combination of MEK1 and MEK2 (MEK1/2) siRNA and incubated at 37 °C for 72 h. At 72 h post-transfection, siRNA-transfected cells were either (**a**) infected with JCPyV (MOI: 1 FFU/cell) at 37 °C for 1 h and then fed with cMEM for 72 h or (**b**,**c**) processed for ICW analysis of MEK1/2 knockdown using a MEK1/2-specific antibody (green) and CellTag control (red). (**a**) Infected cells were fixed and stained to analyze nuclear JCPyV VP1 expression. Data are representative of the mean percentage of JCPyV VP1+ cells per 10x visual field normalized to the siRNA control cells (100%) for triplicate samples (representative of three independent experiments). Error bars = SD. (**b**) MEK1/2 protein expression was measured for control- and experimental-siRNA treatments by ICW analysis, and (**c**) signal intensity values were quantified per the calculation [(protein/CellTag) × 100] as determined through ImageJ analysis. Boxes represent the distribution of the data for three independent experiments; box midline represents the median. Whiskers represent values 1.5 times the distance between the first and third quartiles (inter-quartile range). Student’s *t*-test was used to determine the statistical significance. *, *p* < 0.05.

The ERK1/2 proteins have been previously shown to play an essential role in facilitating viral infection [18,24,25]. Raf protein knockdown through siRNA treatment reduced JCPyV infectivity (Figure 1), suggesting that the MAPK-ERK pathway is activated through Raf during JCPyV infection. Raf and ERK are linked by MEK, which directly phosphorylates ERK [26,27]. MEK isoforms 1 and 2 are dual-specificity kinases, activating threonine and tyrosine residues on both ERK1 and ERK2 [26]. Interestingly, ERK1/2 are currently the only known substrates of MEK, highlighting its crucial role in both activating ERK and facilitating overall MAPK-ERK signaling. Previous studies have investigated the impacts of the MEK chemical inhibitors PD98059 and U0126 on JCPyV infection and found that upon inhibition of MEK, viral infectivity was significantly reduced, suggesting a pivotal role for MEK1/2 induction of ERK1/2 activity in facilitating viral infection [23,24,25]. To further define the role of MEK1/2 in JCPyV infection, SVG-A cells were transfected with siRNAs specifically targeting MEK1/2 or a control. Cells were subsequently infected with JCPyV, and infectivity was scored based on the presence of nuclear VP1 expression. At 72 hours postinfection (hpi), cells treated with MEK1/2 siRNA demonstrated an ~80% decrease in infectivity in comparison to control siRNA-treated cells (Figure 2A). MEK1/2 protein expression was reduced by an ~60% as measured by ICW analysis (Figure 2B,C). These data suggest that MEK1 and 2 are necessary for viral infection, in correlation with previously published work [24,25].

### 2.3. JCPyV Induces MEK Activaiton upon Infection

Previous research has shown that JCPyV infection induces phosphorylation of ERK1/2 at early time-points during viral infection, occurring at 15 min post-infection [18,24,44]. Activation of ERK1/2 is facilitated directly through dual-phosphorylation by MEK during canonical signaling of the MAPK-ERK cascade [27,45]. ERK1/2 proteins are currently the only identified substrates of the dual-specificity kinase MEK [24], and thus MEK may also play an essential role in promoting JCPyV infection (Figure 2). To determine if JCPyV infection activates MEK1/2 activity following viral challenge, SVG-A cells were either mock infected (treated with medium lacking virus) or infected with JCPyV, fixed, and subsequently analyzed using ICW assay to quantitate levels of phosphorylated MEK1/2 (pMEK) during the early stages of infection [25].

**Figure 3:**
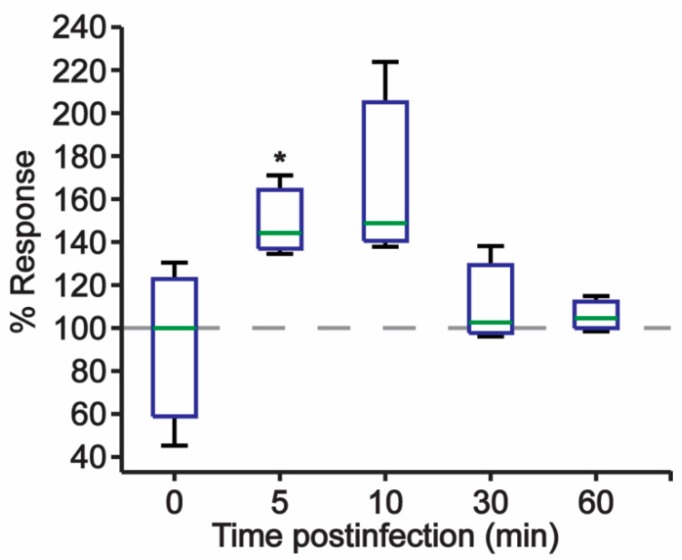
Quantitation of MEK phosphorylation during JCPyV infection. SVG-A cells were mock infected (cMEM only) or infected with JCPyV (MOI: 1 FFU/cell). Cells were then fixed at the indicated timepoint and stained with antibodies targeting phosphorylated MEK1/2 (pMEK1/2) and CellTag (control) for ICW analysis using a LICOR imaging system. The percentage of pMEK1/2 was quantitated by ICW signal intensity values for triplicate samples per the calculation [(pMEK/Cell Tag) × 100] as determined through ImageJ analysis and normalized to mock-infected samples (100%, grey lines). Data are representative of three independent experiments. Boxes represent the distribution of the data; box midline represents the median (green). Whiskers represent values 1.5 times the distance between the first and third quartiles (inter-quartile range). Student’s *t*-test was used to determine the statistical significance. *, *p* < 0.05.

At timepoints as early as 5 and 10 mins post infection, pMEK levels were increased in JCPyV-infected samples in comparison to mock-infected samples, and phosphorylation levels declined over the course of 1 h (Figure 3). These findings demonstrate that JCPyV infection induces MEK1/2 activity at timepoints congruent with ERK activation during JCPyV challenge [18,24], suggesting that JCPyV infection increases levels of pMEK, which can result in increased activation of ERK. These data demonstrate that JCPyV infection utilizes multiple proteins within the MAPK-ERK pathway to promote ERK phosphorylation and overall infectivity.

### 2.4. Gene Expression of Multiple MAPK-ERK-Associated Proteins and Transcription Factors Are Upregulated during JCPyV Infection

Inhibition of essential MAPK-ERK proteins Raf, MEK, and ERK reduces JCPyV infection (Figure 1 and Figure 2) [25], further indicating that JCPyV relies on the MAPK-ERK pathway to promote infection of host cells [18,24]. However, the impact of this cascade on the numerous potential downstream targets in the MAPK-ERK pathway is uncharacterized. Like most cellular signaling mechanisms, the MAPK-ERK cascade is an exceedingly complex network of proteins that interacts with a multitude of different substrates, engages in cross-talk with other pathways, and ignites feedback loops that regulate further signaling propagation [26,27]. Ultimately, these downstream effects can impact mitogenic induction of cellular processes like growth and proliferation [26,27], both of benefit to a virus that utilizes host-cell machinery in order to facilitate genome replication. In the context of JCPyV infection, ERK has been identified as a key regulator of JCPyV viral gene transcription [24], leading to the hypothesis that JCPyV-induced ERK activation drives the activation of host-cell transcription factors. Several transcription factors have been associated with canonical MAPK-ERK pathway including Elk-1, cJun, cMyc, cFos, etc. [46]. Due to the potential MAPK-ERK-regulation of transcription factors and viral reliance on host-cell transcription factors to drive transcription of viral genes, a global qPCR MAPK array was employed to investigate whether gene expression of MAPK-specific transcription factors was influenced during JCPyV challenge (Table 1).

SVG-A cells were infected with JCPyV or mock infected, RNA was harvested at 24 hpi, and cDNA was generated and analyzed for the relative gene expression of MAPK-ERK-associated genes and transcription factors at 24 hpi, based on previous findings that MAPK activation regulated transcription of viral early genes at 48 hpi [24]. Interestingly, several transcription factors were upregulated in comparison to mock-infected samples at 24 hpi including cMyc, Elk-1, SMAD4, and cJun (Table 1), suggesting that JCPyV positively upregulates MAPK-ERK-associated transcription factors during infection. Raf, MEK, and ERK genes were also upregulated following JCPyV challenge as well as other MAPK-associated proteins and transcription factors (Table 1). Interestingly, other groups have identified a portion of these specific transcription factors, including NFAT4 and p53, as necessary factors for facilitating JCPyV infection [35,47], and thus served as internal experimental controls. Further, genes associated with other MAPK pathways were upregulated, including MAPK13 (p38 MAPK) and SMAD4, which can be activated through crosstalk of the p38 MAPK and MEK-ERK pathway [23,48]. Together, these data suggestively link the utilization of these proteins during infection to viral activation of the MAPK-ERK cascade.

### 2.5. JCPyV Induces Altered Nuclear Localization of MAPK-ERK-Associated Transcription Factors

The upregulation of multiple MAPK-ERK-associated transcription factors during JCPyV infection (Table 1) suggests that these transcription factors may play a role in promoting viral gene transcription through MAPK-ERK signaling. Interestingly, regulation of ERK is heavily dependent on its spatial localization within the cell, as downstream effectors of the MAPK-ERK pathway are often spatially and temporally regulated in response to stimuli, facilitating their subsequent activation and response [27,41]. As this spatial regulation of ERK occurs during prototypical MAPK signaling [28], JCPyV infection may influence the localization of activated ERK to specific cellular compartments, including the nucleus, to regulate necessary host-cell transcription factors to promote infection. Transcription factor targets of activated ERK include cMyc and SMAD4, which are activated upon stress or growth factor stimulation [23,27] and, intriguingly, have been demonstrated to be required for JCPyV infection [23,37]. Additionally, MAPK pathway-regulated transcription factor cJun was also upregulated at 24 hpi (Table 1), and cJun has been previously reported to be unchanged during early time points in a single replication cycle [49] yet positively regulated during long-term infections [34]. As MAPK-regulated transcription factors are upregulated during JCPyV infection (Table 1), MAPK-ERK activation induced by JCPyV may facilitate transcription factor accumulation in the infected host-cell nucleus.

**Figure 4:**
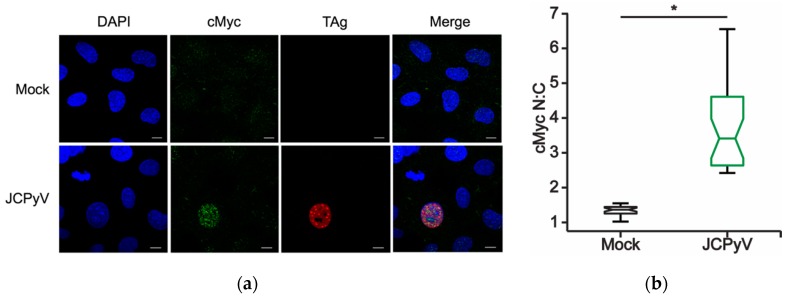

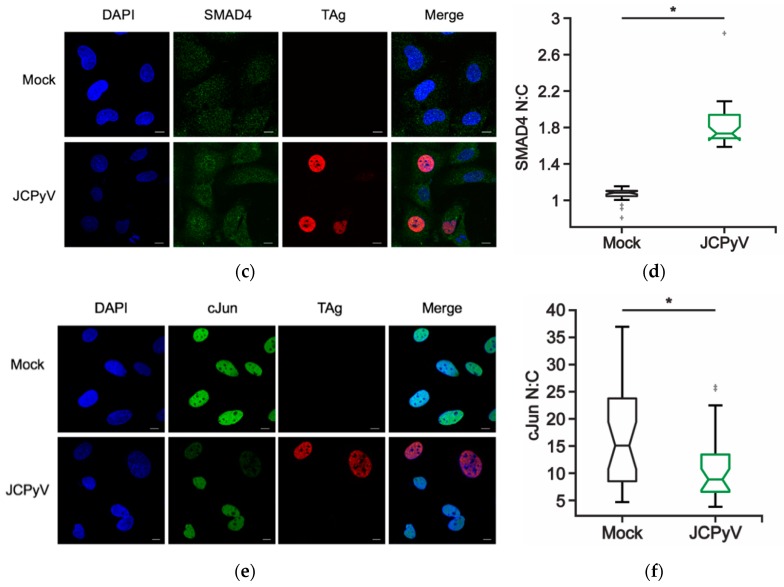
MAPK-regulated transcription factor localization during JCPyV infection. SVG-A cells were infected with JCPyV (MOI: 2 FFU/cell) and incubated in cMEM at 37 °C for 48 h, then fixed and processed for confocal imaging. Representative confocal images (60x) demonstrating (**a**) cMyc, (**c**) SMAD4, and (**e**) cJun (green) cellular localization patterns during mock or JCPyV infection, as measured by TAg expression (red). Cell nuclei stained with DAPI (blue). Scale bars = 10 μm. Boxplots of N:C ratios for (**b**) SMAD4, (**d**) cMyc, or (**f**) cJun quantified from confocal images of 30 individual cells. Whiskers represent values 1.5 times the distance between the first and third quartiles (inter-quartile range). Boxplot notches denote the 95% confidence interval around the median. Data are representative of N:C ratios from three independent experiments. Wilcoxon sum rank test was used to determine statistical significance. * *p <* 0.05.

To investigate the temporal localization of MAPK-regulated transcription factors in response to JCPyV infection, SVG-A cells were infected with JCPyV or mock infected then fixed and stained for both the viral early gene product TAg and a host-cell transcription factor target identified in the MAPK array: cMyc, SMAD4, or cJun. Infected cells expressing TAg were assessed for nuclear versus cytoplasmic localization for the transcription factors of interest in comparison to their mock-infected cell counterparts at 48 hpi. Through ImageJ analysis [50], nuclear and cytoplasmic regions of interest (ROIs) were established to quantitate the signal intensity of the host protein of interest between mock- and JCPyV-infected cells in both the nucleus and cytoplasm of single cells [50]. Through the generation of nuclear:cytoplasmic (N:C) ratios, potential shifts in localization patterning of these transcription factors during mock or JCPyV infection were quantified. In comparison to mock-infected cells, SVG-A cells challenged with JCPyV demonstrated increased N:C ratios for both SMAD4 and cMyc in TAg-expressing cells (Figure 4A–D). These findings suggest that JCPyV infection promotes an increase in the nuclear localization of both cMyc and SMAD4 transcription factors during the infectious process. Conversely, the relative N:C ratio of cJun localization in JCPyV-infected cells decreased in comparison to mock-infected cells, indicating that JCPyV infection does not induce nuclear localization of cJun at 48 hpi (Figure 4F). These findings corroborate established findings that report cJun nuclear protein expression is not significantly enhanced in JCPyV-infected cells during the initial replication cycle, yet cJun expression is increased in subsequent rounds of infection [34]. However, cJun gene expression was upregulated in the MAPK array at 24 hpi (Table 1), suggesting that cJun may be differentially regulated at various times post infection, which aligns with previous reports [34]. Together, these findings demonstrate that JCPyV infection alters the normal expression levels and localization patterns of MAPK-associated transcription factors including cMyc and SMAD4, potentially highlighting a resultant mechanism of JCPyV-induced MAPK activation for viral transcription.

### 2.6. MAPK-ERK Signaling Regulates cMyc and SMAD4 Cellular Localization Patterns

JCPyV infection induces ERK phosphorylation early in the infectious process [18,24,25]; however, JCPyV requires this kinase activity to facilitate later steps in the viral lifecycle, including viral gene transcription [24]. Further, the global MAPK qPCR array revealed changes in gene expression of several MAPK-associated transcription factors and associated proteins upon JCPyV challenge (Table 1). These identified transcription factors, including cMyc and SMAD4, were also found to have an increased N:C intensity upon JCPyV infection, suggesting that these transcription factors both elicit a response to MAPK-induced signaling. 

**Figure 5:**
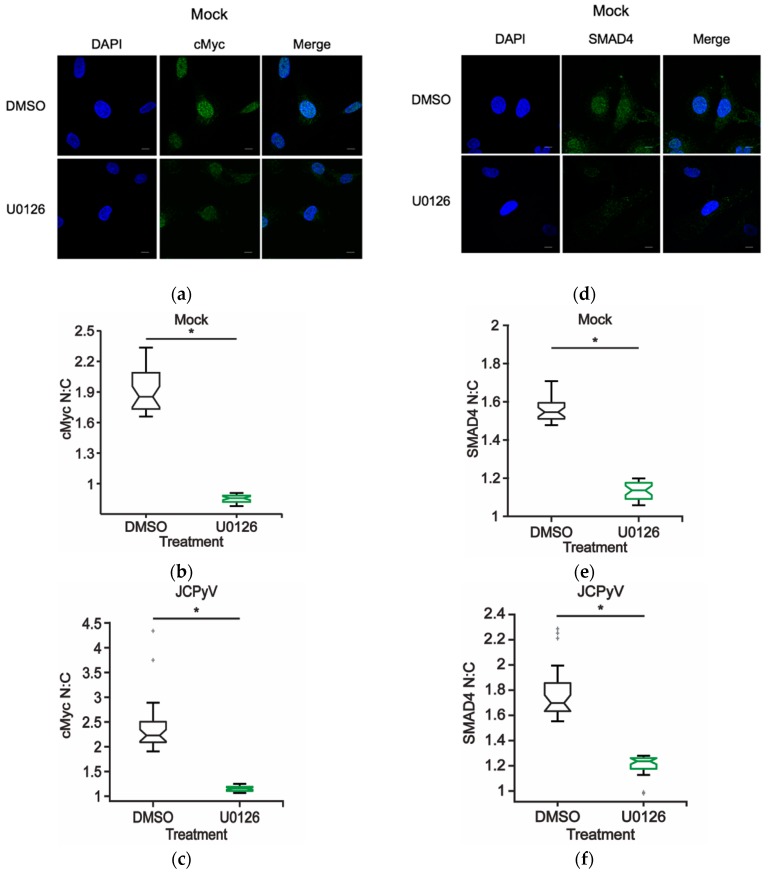
ERK inhibition reduces nuclear localization of MAPK-associated transcription factors during JCPyV infection. SVG-A cells were treated with U0126 (10 μM) or DMSO (vehicle control) in cMEM and incubated at 37 °C for 48 h, then fixed and processed for confocal imaging. Representative confocal images demonstrating (**a**) cMyc and (**d**) SMAD4 (green) cellular localization patterns during DMSO- or U0126- treatment (10 μM) in mock-infected cells (100x). Cell nuclei stained with DAPI (blue). Scale bar = 10 μM. Boxplots of N:C ratios of (**b**) cMyc and (**e**) SMAD4 quantified from confocal images (60x) of 30 individual cells of DMSO- and U0126-treated mock-infected cells (60x). (**c**,**f**) SVG-A cells were treated with either U0126 (10 μM) or DMSO (vehicle control) containing cMEM for 1 h, infected with JCPyV (MOI: 2 FFU/cell), and incubated in cMEM at 37 °C for 1 h. At 1 hpi infected cells were fed with appropriately treated media (DMSO or U0126), incubated at 37 °C for 48 h, then fixed and processed for confocal imaging. Boxplots of N:C ratios for (**c**) cMyc and (**f**) SMAD4 quantified from confocal images of 30 individual cells of DMSO- and U0126-treated cells infected with JCPyV for 48 h. Whiskers represent values 1.5 times the distance between the first and third quartiles (inter-quartile range). Boxplot notches denote the 95% confidence interval around the median. Data are representative of N:C ratios from three independent experiments. Wilcoxon sum rank test was used to determine statistical significance. *, *p* < 0.05.

To determine if ERK signaling directly influences transcription factor localization in glial cells, SVG-A cells were treated with either DMSO or U0126 for 48 h and cells were subsequently assessed for cMyc and SMAD4 protein localization using confocal imaging to define the nuclear versus cytoplasmic localization patterns (N:C ratios) (Figure 5). In comparison to DMSO-treated cells, SVG-A cells treated with the MEK1/2 inhibitor U0126 [24], demonstrated decreased N:C ratios for both cMyc (Figure 5A,B) and SMAD4 (Figure 5D,E). Moreover, to determine whether ERK inhibition hindered nuclear localization of cMyc and SMAD4 in the presence of JCPyV, DMSO- and U0126-treated SVG-A cells were infected with JCPyV, and N:C ratios were analyzed using Image J (Figure 5C,F). Nuclear localization of cMyc and SMAD4 was reduced in ERK-inhibited cells in contrast to the increase in nuclear localization during JCPyV infection when cells are not treated with U0126 (Figure 4). The reduction in SMAD4 activation due to ERK inhibition with U0126 is in line with previous results that demonstrate that JCPyV activation of SMAD4 is regulated through the ERK pathway and blocked by the MEK inhibitor U0126 [23]. These findings suggest that JCPyV infection induces cellular localization of cMyc and SMAD4 through a mechanism that is regulated through the MEK-ERK pathway (Figure 5C,F). The results described herein demonstrate that ERK activity may promote the accumulation of both cMyc and SMAD4 in the host-cell nucleus during canonical MEK-ERK signaling and that inhibition of ERK prevents JCPyV-induced accumulation of these necessary transcription factors within the host-cell nucleus.

## 3. Discussion

Previous studies have identified a critical role for ERK, a key member of the MAPK pathway, in promoting JCPyV infection. However, the function of the MAPK signaling cascade and the pathway responsible for ERK activation during infection had yet to be elucidated. The findings presented herein have identified that the additional core kinases that comprise the MAPK-ERK pathway, Raf and MEK, each play necessary roles in promoting JCPyV infection. The initial kinase of this cascade, Raf, specifically B-Raf and C-Raf, was shown to be necessary for infection (Figure 1), suggesting that JCPyV-induced activation of ERK is regulated through the prototypical MAPK signaling cascade. The necessity of the downstream kinase MEK was also characterized through the siRNA knockdown of MEK1/2 in SVG-A cells. Upon protein knockdown of MEK1/2, the percentage of infected cells was significantly diminished, suggesting that MEK also plays a vital role in successful JCPyV infection (Figure 2). Further, phosphorylation levels of MEK1/2 were activated at 5–10 mins post-infection (Figure 3), suggesting that ERK activation upon JCPyV infection requires the initiation of canonical MAPK-ERK phosphorylation events. Together, these findings demonstrate the JCPyV requires multiple facets of the MAPK-ERK pathway to promote productive infection in glial cells. 

The activation of Raf to initiate the MEK-ERK cascade typically emanates from extracellular signals such as ligands or growth factors activating RTKs or G-protein coupled receptors (GPCRs). Interestingly, JCPyV requires the GPCR serotonin (5-HT_2_) receptors for viral entry and infection [16,19]. Thus, it is plausible that activation of the 5-HT_2_ receptors leads to activation of Ras, which in turn then recruits and activates Rafs. Inhibition of B-Raf and C-Raf with specific inhibitors or with targeted siRNAs (Figure 1) [25] leads to reduced JCPyV infection. Interestingly, the C-Raf isoform is the most ubiquitously expressed, and B-Raf is expressed in neuronal tissues and in glial cells [51,52]. A-Raf is expressed in urogenital tissues [51], and given that JCPyV can also infect the urogenital tract and inhibition of ERK in primary kidney cells reduces JCPyV infection [25], it is possible that A-Raf may play a role in MEK-ERK activation in these tissues during infection. All Raf isoforms were upregulated following JCPyV infection (Table 1), suggesting that all isoforms are present within SVG-A cells. However, B-Raf and C-Raf play a predominant role in activating MEK1/2 [41], while A-Raf functions to activate B-Raf and stabilize B-Raf:C-Raf complexes in some cell types leading to MEK-ERK activity [53,54]. Therefore, cell-type and tissue-specific regulation of Raf and MAPK signaling in JCPyV infection and the potential receptor-induced activation require further exploration. 

Signal transduction through the MAPK-ERK cascade ultimately stimulates a cellular response through the activation of multiple effector proteins including transcription factors [27,28,45]. Additionally, JCPyV requires MAPK-ERK signaling to promote infection, particularly in order to promote viral gene transcription [25]. Data presented herein suggest that JCPyV may require the activation of the MAPK-ERK cascade to target specific proteins within this pathway to facilitate productive infection. Using a global MAPK qPCR array, multiple genes associated with the activation of the MAPK-ERK pathway were identified to be upregulated during JCPyV infection (Table 1). For instance, Kinase Suppressor of Ras 1 (KSR1) was upregulated following infection, and KSR1 encodes for a scaffolding protein which can localize to the plasma membrane and complex with B-Raf and MEK, promoting the phosphorylation of Rafs and activation of ERK1 and ERK2 [55]. In addition, downstream targets of the MEK-ERK pathway were activated including transcription factors (Table 1). Previous studies have identified numerous host-cell transcription factors usurped by JCPyV during infection including cMyc, AP-1 (cJun and cFos), NFAT4, SMAD4, YB-1, NF-1, and others [23,34,35,36,37,38,39]. Interestingly, many of these transcription factors are downstream effectors of the MAPK-ERK signaling cascade and were upregulated in the qPCR array (Table 1). Additionally, novel transcription factors not previously reported to regulate JCPyV infection were also upregulated. For instance, MYC Associated Factor X (MAX), which encodes for the protein Max, which can bind to cMyc and act as a transcriptional activator [56], is upregulated following JCPyV infection along with cMyc (Table 1). The increased gene expression levels identified using the MAPK global qPCR array suggests that numerous MAPK-regulated genes are upregulated during JCPyV infection, yet their specific functions in JCPyV infection remain to be characterized. 

As the spatiotemporal regulation of canonical MAPK signaling plays a critical role in targeting specific downstream proteins like transcription factors, the localization patterns of identified transcription factors were measured during viral infection. Under normal cellular conditions, both cMyc and SMAD4 can localize to cytoplasmic and nucleoplasmic regions within the cell, while cJun is highly retained in the nucleus [57,58]. The nuclear to cytoplasmic ratios for both cMyc and SMAD4 at 48 hpi indicate that these transcription factors localize in the nucleus over the cytoplasm of infected cells (Figure 4). In contrast, cJun N:C ratios were decreased in JCPyV infected samples in line with previous findings of decreased cJun expression during a single round of JCPyV replication [34]. As cMyc and SMAD4 nuclear accumulation relies on proper ERK signaling (Figure 5), the direct stimulation of this pathway during viral challenge may further highlight the role of the MAPK-ERK signaling cascade in JCPyV transcription. 

JCPyV infection has been previously shown to induce alterations to transcription factor localization patterning of SMAD4 [23]. Ravichandran et al. have shown that upon treatment with transforming growth factor-β1 (TGF-β1), the kinase MEK is activated, and the SMAD2/4 complex translocates from the cytoplasm into the nucleus, thereby enhancing JCPyV replication [23]. Additionally, they demonstrated that increased JCPyV infection through TGF-β1 signaling was activated specifically through ERK, as MEK inhibitors reduced the activation of SMAD4 [23]. SMAD4 activation is usually regulated through TGF-β1-mediated stimulation of the p38 MAPK pathway [48]. Interestingly, the global qPCR MAPK array revealed that p38 MAPK was upregulated upon JCPyV infection at 24 hpi (Table 1) which implies that JCPyV may upregulate this MAPK pathway during infection. However, Ravichandran et al. reported that TGF-β1-mediated activation of SMAD4 in JCPyV infection was reduced by inhibition of MEK with U0126 [23]. The results presented herein corroborate previous findings demonstrating that nuclear accumulation of SMAD4 was dampened upon treatment with U0126 (Figure 5), suggesting that MAPK-ERK signaling facilitates SMAD4 nuclear localization. Thus, JCPyV could induce a stress response in infected cells resulting in crosstalk between the MEK-ERK MAPK pathway and the p38 MAPK pathway, resulting in activation of SMAD4, which is necessary for infection, yet components of the p38 MAPK pathway may be activated but not play a direct role in JCPyV replication. These data further clarify the activation and spatial and temporal patterning of SMAD4 during JCPyV infection. Additionally, these findings emphasize that ERK activity is required for the localization of the key transcription factors cMyc and SMAD4 during JCPyV infection. The connection between the nuclear localization of both cMyc and SMAD4 and MAPK-ERK activity suggests that JCPyV infection may specifically upregulate this robust signaling pathway in order to promote viral gene transcription.

The MAPK-ERK pathway plays critical roles in promoting important cellular processes such as growth, differentiation, proliferation, and pro-survival signaling. These requisite cellular activities are also targeted by most human DNA viruses during the infectious process, as manipulating these mechanisms is key to promoting successful viral infection of the host. With a small genome size, JCPyV only encodes for six protein-coding genes, necessitating that each of the resultant viral proteins act in multiple capacities to promote infection. Capitalizing on endogenous host-cell mechanisms that are responsible for multiple cellular fates is one way that viruses can effectively function with a such limited genetic toolkit. Indeed, many human DNA viruses utilize MAPK-ERK signaling, often to facilitate viral replication, dysregulate the cell-cycle, alter host immune responses, or even promote cell survival [44].

## 4. Materials and Methods

### 4.1. Cell Types, Virus Strains, Reagents, and Antibodies

SVG-A cells [59] were cultured in Minimum Essential Medium (MEM) (Corning) containing 10% fetal bovine serum (FBS), 1% penicillin/streptomycin (P/S) (Mediatech, Inc., Corning, NY, USA) and 0.2% Plasmocin prophylactic (Invivogen) (cMEM). Cells were grown in a humidified incubator at 37 °C with 5% CO_2_. SVG-A cells were generously provided by the Atwood laboratory (Brown University) and authenticated by ATCC. JCPyV strain Mad-1/SVEΔ (provided from the Atwood laboratory (Brown University)) was purified as described [60] and used for infectivity experiments as indicated. The MEK(1/2) chemical inhibitor U0126 (Cell Signaling Technology (CST)) was reconstituted in DMSO and was used at the indicated concentration of 10 μM for 48 h. DMSO served as a volume-specific vehicle control.

Antibodies used for detection of viral proteins include PAB597 (provided by Ed Harlow) and PAB962 (provided by the Tevethia laboratory) hybridoma supernatants that produce a monoclonal antibody against JCPyV VP1 or large T Antigen (TAg), respectively. Antibodies used for ICW analysis of protein knockdown or phosphorylation include: B-Raf (1:300, CST), C-Raf (1:500, CST), phospho MEK1/2 (1:500, CST), and MEK1/2 (1:500, CST). Additionally, CellTag 700 (Cell normalization stain) (1:500, LI-COR), as well as, secondary LI-COR 800 anti-rabbit or anti-mouse antibodies (1:10,000, LI-COR) were used for ICW protein detection analyses. Antibodies and reagents used for confocal immunofluorescence imaging include: cJun (1:800, CST), SMAD4 (1:500, CST), cMyc (1:500, CST) and DAPI nuclear counterstain (1:1000, Thermo Fisher Scientific).

### 4.2. siRNA Treatment

SVG-A cells were plated to 50% confluency in 12 well plates (Greiner Bio-One, Kremsmünster, Austria) in FBS-containing media, lacking antibiotics. Cells were transfected with a non-targeting unconjugated scrambled (control) (CST), B-Raf (CST), C-Raf (CST), or a combination of MEK1 and MEK 2 (CST) siRNAs using RNAiMax (Invitrogen) per the manufacturer’s instructions. In brief, RNAiMax transfection reagent and siRNAs (30 pmol/siRNA/well) were prepared in incomplete media (iMEM) (lacking FBS and antibiotics), combined, and incubated at RT for 10 min. MEK1 and MEK2 siRNAs (15 pmol/each) were combined for a final concentration of 30 pmol/siRNA/well. siRNA complexes were added to SVG-A cells and incubated at 37 °C for 72 h. At 72 h post-transfection cells were either infected with JCPyV (MOI = 1 FFU/cell, virus lysate stock) at 37 °C for 1 h or fixed with 4% PFA, washed with 1X PBS three times, and analyzed to confirm siRNA protein knockdown by ICW. Infected cells were fed with cMEM and incubated at 37 °C for 72 h. At 72 hpi, cells were fixed with 4% PFA, washed with 1X PBS three times, and stained for VP1 by indirect immunofluorescence and quantitation of viral infection as described.

### 4.3. Indirect Immunofluorescence Detection and Quantitation of Viral Infection

Following PFA fixation, SVG-A cells were permeabilized with 1X TBS- 1% Triton X-100 for 15 min and were then incubated with 10% goat serum (Vector Labs, Burlingame, CA, USA) in 1X TBS-T at RT for 1 h while rocking. Cells were stained with either PAB597 (VP1 Ab, 1:40) in 1X TBS-T at 37 °C for 1 h. Cells were then washed with 1X TBS-T and incubated with an anti-mouse Alexa Fluor 488 antibody (Thermo Fisher Scientific, Waltham, MA, USA) at 37 °C for 1 h and nuclei were counter stained with DAPI at RT for 5 min (Thermo Fisher Scientific, Waltham, MA, USA). Using a Nikon Eclipse Ti epifluorescence microscope (Micro Video Instruments, Inc., Avon, MA, USA), the number of VP1-expressing cells per 10x visual field for 5 visual fields (per well) was quantitated. Percent infection was determined by dividing the number of infected cells per visual field by the total number of DAPI-positive nuclei per visual field as previously described [24]. As indicated, the average percent infection was normalized to the indicated control (100%).

### 4.4. ICW Analysis of Phosphorylation of MEK1/2 during JCPyV Infection

SVG-A cells were plated to 70% confluency in 96 well plates. Cells were either mock-infected (cMEM only) or infected with purified JCPyV (MOI = 1 FFU/cell) in cMEM at 37 °C for 0, 5, 10, 30, or 60 min. At indicated timepoints, cells were fixed in 4% PFA and washed in 1X PBS. Following PFA fixation, cells were incubated with 1X TBS-1% Triton X-100 at RT for 15 min to permeabilize. Cells were then incubated with TBS Odyssey Blocking Buffer (LI-COR, Lincoln, NE, USA) at RT for 1.5 h while rocking. Cells were stained with primary antibody pMEK1/2 (1:500) in TBS Odyssey Blocking Buffer (LI-COR) at 4 °C O/N while rocking. After incubation with the primary antibody, cells were washed with 1X TBS-T and then incubated with the rabbit LI-COR 800 secondary antibody (1:10,000) and CellTag 700 (1:500) at RT for 1 h while rocking. Cells were then washed with 1X TBS-T three times and aspirated to remove all liquid prior to scanning. Using a LI-COR Odyssey CLx, plates were scanned for 700 and 800 nm channel intensities. In addition, for detection of protein knockdown, following siRNA and PFA fixation, SVG-A cells were washed 3 times with 1X PBS, and permeabilized with 1X TBS-1% Triton X-100 at RT for 15 min. Cells were then incubated with TBS Odyssey Blocking Buffer (LI-COR) at RT for 1.5 h while rocking. Cells were stained with primary antibodies for B-Raf (1:300), C-Raf (1:500), or MEK1/2 (1:500) in TBS Odyssey Blocking Buffer (LI-COR) at 4 °C O/N while rocking. After incubation with the primary antibody, cells were washed with 1X TBS-T and then incubated with the rabbit LI-COR 800 secondary antibody (1:10,000) and CellTag 700 (1:500) at RT for 1 h while rocking. Cells were then washed with 1X TBS-T three times and aspirated to remove all liquid prior to scanning. Plates were read at a resolution of 42 μM, at medium quality, and a 3.0 mm focus offset. After scanning, channels were aligned using the Image Studio software version 5.2 (LI-COR, Lincoln, NE, USA) equipped with the In-cell Western module. After scanning, the ICW analysis grid (Image Studio) was applied to the plate image to outline each well and images were then processed using Image J (NIH) [61] as previously described [25]. Following quantitation, % response was determined using R (version 3.6.1) equipped with RStudio (version 1.2.1335, 2019, Integrated development of RStudio, Inc., Boston, MA, USA) for three independent experiments.

### 4.5. Global RT^2^ MAPK-ERK Profiler qPCR Array

SVG-A cells were mock infected or infected with purified JCPyV (MOI: 1 FFU/cell) and incubated at 37 °C for 1 h. Cells were fed with cMEM and incubated at 37 °C for 24 h. At 24 hpi, RNA was extracted from each sample with the SingleShot Cell Lysis kit (Bio-Rad, Hercules, CA, USA) per manufacturer’s protocol. Harvested RNA from each sample was converted to cDNA using iScript Reverse-Transcription Supermix (Bio-Rad, Hercules, CA, USA) with 1 μg of RNA per reaction. cDNA prepared from mock-infected and JCPyV-infected samples was combined with the RT^2^ SYBR Green qPCR Master Mix (Qiagen), aliquoted into individual RT^2^ Profiler PCR Array plates (Qiagen), then wells were sealed and centrifuged at 168× *g* for 1 min. PCR array plates were thermal cycled per the manufacturer’s instructions using a Bio-Rad CFX96 Real-Time System version 3.1 (Bio-Rad CFX Manager, Hercules, CA, USA). Calculation of the ΔCT for genes for interest (GOI) were determined using the average of three designated housekeeping genes (HKG) on each plate (GOI CT − HKG CT). Calculation of ΔΔCT for JCPyV-infected samples was determined in comparison to mock-infected samples (JCPyV GOI ΔCT − Mock GOI ΔCT) for relevant GOIs. The average fold change of GOI transcript levels between mock-infected and JCPyV-infected cells was performed using 2^−ΔΔCT^ of average calculated GOI ΔΔCT values. Average fold change of GOIs was determined by using samples from three independent experiments.

### 4.6. Detection of Host and Viral Proteins during JCPyV Infection with Confocal Imaging

SVG-A cells were plated to 50% confluency in 14 mm glass bottom dishes (MatTek, Ashland, MA, USA) in cMEM containing 2% FBS (2% cMEM) and were incubated at 37 °C overnight. Cells were either mock infected or infected with purified JCPyV (MOI = 2 FFU/cell) in 2% cMEM at 37 °C for 1 h. Cells were then fed with 2% cMEM and incubated at 37 °C for 48 h. At the indicated timepoint, both mock and infected samples were fixed and processed for confocal imaging.

### 4.7. Analysis of Host and Viral Protein Localization in Mock- and JCPyV-Infected Cells during MEK Inhibition

Mock-infected samples: SVG-A cells were plated to 50% confluency in 14 mm glass bottom dishes in 2% cMEM and were incubated at 37 °C O/N. Cells were then treated with 2% cMEM containing DMSO or U0126 (10 μM) at 37 °C for 48 h. At 48 h, samples were fixed and processed for confocal imaging. 

JCPyV-infected samples: SVG-A cells were plated to 50% confluency in 14 mm glass bottom dishes in 2% cMEM and were incubated at 37 °C O/N. Cells were then treated with 2% cMEM containing DMSO or U0126 (10 μM) at 37 °C for 1 h. After 1 h pretreatment, both DMSO- and U0126-treated cells were infected with purified JCPyV (MOI = 2 FFU/cell) in 2% cMEM at 37 °C for 1 h. Cells were then fed with the appropriate DMSO- or U0126-treated media for 48 h. At 48 h, samples were fixed and processed for confocal imaging.

### 4.8. Confocal Imaging and Analysis of Host and Viral Proteins

Cells were fixed in 4% PFA and washed in 1X PBS three times. Following fixation, samples were permeabilized with 1X TBS-1% Triton X-100 at RT for 15 min and were then incubated with 10% goat serum (Vector Labs) in 1X TBS-T at RT for 1 h while rocking. Cells were stained with antibodies for PAB962 for viral TAg (1:3) and host-cell proteins cJun (1:800), cMyc (1:500), or SMAD4 (1:500) in 1X TBS-T at 37 °C for 1 h. Cells were then washed with 1X TBS-T and incubated with an anti-rabbit Alexa Fluor 488 antibody (Thermo Fisher Scientific) at 37 °C for 1 h, and nuclei were counter stained with DAPI (1:1000).

Cells were visualized using a laser-scanning confocal microscope (model IX81, Olympus America, Inc.) equipped with 405–633 nm laser lines using a 60X or 100X, 1.42NA oil immersion lens (Olympus, Center Valley, PA, USA). Images were acquired using the Olympus FluoView application suite version 04.01.01.05 (Olympus, Center Valley, PA, USA). For each sample type, 30 cells were imaged per replicate across three independent experiments for a total of 90 cells per sample type. Images were analyzed for nuclear:cytoplasmic (N:C) ratios for host-proteins of interest with ImageJ.

### 4.9. ImageJ Quantitation of Nuclear:Cytoplasmic Ratio of Host Proteins

Confocal images were processed for host-protein N:C ratios among mock and infected samples in ImageJ as described [50]. In brief, maximum intensity projections of the DAPI (405), host-protein (488), and TAg (594) (if applicable) channels were generated and merged within a single sample. The background was subtracted from each image using the rolling ball radius method. The DAPI (405) nuclear stain was used to generate a nuclear mask of cells within the image excluding cells on the periphery. The DAPI image threshold was adjusted, converted to a binary image, and analyzed to generate nuclear regions of interest (ROIs). This nuclear mask was then applied to the host-protein (488) channel, and the nuclear ROIs were measured, generating the mean nuclear intensity value of the host-protein of interest. The nuclear mask was then dilated; new regions of interest were generated and were then applied to the host-protein channel to define the mean cytoplasmic intensity of the host-protein of interest. The N:C ratios were then calculated as described [50]. Boxplots were generated using MATLAB and Statistics Toolbox release R2018a (The MathWorks, Inc., Natick, MA, USA).

### 4.10. Statistical Analyses

Experiments were performed in triplicate containing a minimum of triplicate samples or a minimum of 30 samples for microscopy analysis. Data was analyzed using Matlab and Statistics Toolbox to determine the appropriate statistical test for each experiment. Samples that were normally distributed were analyzed using a two-sample, student’s t test assuming either equal unequal variances (when appropriate) to compare the means for at least triplicate samples. *p*-values < 0.05 were considered statistically significant. The Anderson–Darling test was used to determine if the populations were normally distributed and variation between populations was determined using the F Test for Equal Variance. The Wilcoxon rank sum test was used in populations that were not normally distributed. *p*-values < 0.05 was considered statistically significant.

## Figures and Tables

**Table 1:** Global MAPK qPCR array gene expression analysis of JCPyV-infected cells.

Gene of Interest (Protein)	Relative Fold Change (+)
ARAF (A-Raf)	5.28
BRAF (B-Raf)	3.18
CCND2 (Cyclin D2)	1.30
JUN (cJun)	6.49
ELK1 (Elk-1)	4.51
FOS (Fos)	3.82
KSR1 (Kinase suppressor of Ras 1)	5.36
MAP2K1 (MEK1)	3.62
MAP2K2 (MEK2)	5.62
MAPK1 (ERK1)	4.28
MAPK3 (ERK2)	5.99
MAP2K4 (MAPKK4)	4.06
MAPK13 (p38 MAPK)	8.35
MAX (Myc-associated protein X)	4.79
MEF2C (MEF2C)	7.34
MKNK1 (MAPK Interacting Ser/Thr Kinase)	6.02
MOS (Proto-oncogene Serine/threonine-Protein Kinase mos)	6.52
MYC (cMyc)	4.95
NFATC4 (NFAT4)	14.29
RAF1 (C-Raf)	3.88
SMAD4 (SMAD4)	4.04
TP53 (p53)	5.81
